# A feasibility study of the physiotherapy management of urinary incontinence in athletic women: trial protocol for the POsITIve study

**DOI:** 10.1186/s40814-020-00638-6

**Published:** 2020-07-16

**Authors:** K. Gillian Campbell, Mark E. Batt, Avril Drummond

**Affiliations:** 1 Faculty of Medicine and Health Sciences, University of Nottingham, Nottingham, NG7 2HA UK; 2 Centre for Sports Medicine, Nottingham University Hospitals, Nottingham, UK

**Keywords:** Urinary incontinence, Pelvic floor muscle training, Athletes, Physiotherapy

## Abstract

**Background:**

Urinary incontinence (UI) affects up to 40% of adult women within the UK, and pelvic floor muscle training can be effective as a treatment. The prevalence of UI is higher in athletic women than in their sedentary counterparts, but there is little research into reasons for this or into treatment within this population.

The aim of this study is to investigate the feasibility of conducting a future randomised controlled trial of physiotherapeutic management of UI in athletic women.

**Methods:**

This is a mixed methods study with three distinct but related phases.

Phase 1: Semi-structured interviews with health care professionals in the community will explore current management practices of UI in women and particularly in female athletes in order to inform the control arm of a future study. It will also establish community health care professionals’ understanding of pelvic health physiotherapy.

Phase 2: Athletic and regularly exercising women recruited directly from gyms and sports clubs will undergo a course of physiotherapy to manage UI. This will establish study recruitment, eligibility, consent, attendance, attrition, and data completion rates. It will provide information regarding appropriate clinical venues and outcome measures to use for this patient group.

Phase 3: Semi-structured interviews with purposefully selected participants from phase 2 will investigate participant satisfaction with recruitment procedures, the intervention, outcome measures and the venues. Further, we will collect data regarding the use of a smartphone ‘app’ for adherence and monitoring of home exercises and participants’ beliefs around randomisation in a future study. We will explore the impact of UI on life and sport in more detail.

**Discussion:**

This study will establish the ease and acceptability of recruiting athletic women directly from gyms and sports clubs and identify attrition rates. It will also explore the acceptability of the intervention, clinical venues and outcome measures. Data collected will be used to inform a future randomised controlled trial.

**Trial registration:**

NCT03986411 (clinicaltrials.gov). Registered on 14 June 2019

## Background

Urinary incontinence (UI) is a common problem, with prevalence reported to be as high as 40% of adult women in the UK [1]. UI is defined as involuntary loss of urine [2]. The term includes stress urinary incontinence (SUI) associated with physical exertion, such as sports, or coughing and sneezing, and urgency urinary incontinence (UUI), which is leaking associated with increased urgency or desire to void. Women may experience a combination of these symptoms, that is, mixed urinary incontinence (MUI) [2]. UI is embarrassing and debilitating, affecting all aspects of life. There is evidence that it reduces participation in sport and exercise [3, 4], affects employment, causes absence from the workplace [5], and is detrimental to personal relationships [6]. It is widely accepted that UI can be associated with childbirth, obesity and the ageing process [7]. The primary cause is presumed to be weakness of the pelvic floor muscles (PFM), and the optimal first line in treatment is therefore to improve PFM function with exercises to improve strength, timing and endurance [8, 9]. Evidence supports a supervised pelvic floor muscle training (PFMT) programme for 3 months in the general population [10]. This is recommended in the recent guidelines as first line management of UI by the National Institute for Health Care and Excellence (NICE) [11].

Somewhat surprisingly, the prevalence of UI in young, nulliparous athletes with apparently low risk reveals particularly high rates, ranging from 23–41% [12–15]. UI in athletic women has been reported to be almost double that in a matched group of sedentary individuals [16]. Indeed, an investigation into recreationally active women revealed an even higher occurrence of 49% [17]. This population had a large age range, 18–83, and parity, which may explain the high prevalence in comparison to other studies of younger nulliparous women with fewer risk factors.

There is robust evidence to support pelvic floor muscle training as a treatment intervention in women with UI [18]. In a recent Cochrane review, which included 31 studies and 1837 women from 4 countries, the authors concluded that, in the short term at least, PFMT could effect a cure or improvement in the symptoms of all types of UI [9]. However, studies generally did not follow up treatment effects for more than 1 year, and it was noted that there was a greater improvement rate in those with SUI than other types of UI. Despite the high prevalence of UI in athletic women, there is, however, little research regarding the management of UI within this group [19–21].

It has been suggested that there are two conflicting mechanisms for UI in athletic women: firstly, that athletes have stronger pelvic floors than non-athletes due to the training effect of repeated impact, and secondly, that the repeated challenge of ground reaction forces from running and jumping may instead weaken and stretch the fascia and muscle tissues within the pelvis [22]. In a recent study, comparing continent and incontinent athletes, those that were incontinent were found to have stronger pelvic floor muscles [23].

It is possible that if the pelvic floor muscle is strong and stiff but inextensible, or unable to relax fully, this will cause urinary symptoms. An overactive group of PFM would potentially be unable to react to ground reaction forces adequately, as it is already in a shortened position [24].This may explain why athletes with strong pelvic floors would still present with UI. Increased PFM tension has been noted in cases of increased urinary urgency and frequency with or without associated pelvic pain [25]. Indeed, assessment of some PFM can reveal areas within the tissue that are overactive or ‘tight’ [26] although there is little published data regarding how prevalent this is or whether it is more likely in certain groups of women. It is, however, reasonable to suggest that the PFM in a symptomatic athlete will be dysfunctional as opposed to weak. In this case, it is imperative that the muscles undergo specialist assessment in order to identify the appropriate training and rehabilitation programme for the individual. Standard, prescriptive protocols via leaflets, advising general PFM strengthening without tailored advice, may not afford any real improvement and indeed, could aggravate the symptoms.

Despite the high prevalence of UI in women, fewer than half will present to healthcare professionals for help [1]. This is potentially an even bigger issue in athletes with UI, where 90% of those reporting UI in questionnaires had not previously mentioned their symptoms to anyone [14]. It is important to understand why these women do not seek help and ensure that those that do so are offered effective, evidence-based treatment.

It has been identified that there is a need for high-quality research in this area and specifically randomised clinical trials [21]. Our ultimate aim is to conduct a randomised controlled trial (RCT) to determine whether one to one physiotherapy is an acceptable, cost-effective way to improve the symptoms of UI in athletic women. This study is a first step to investigating the feasibility of conducting such a trial. It will explore treatments currently offered within the community setting to athletic women presenting with UI. Further, it will investigate the acceptability of direct recruitment from gyms and sports clubs and of providing one to one specialist pelvic physiotherapy within this cohort. It will provide information about the consent procedures, outcome measures and retention of participants. This feasibility study will enable us to determine whether conducting a definitive appropriately powered trial is possible.

## Methods

### Study Management

A steering group comprised of the co-investigators, two service representatives and a specialist pelvic health physiotherapist will guide the overall management of the study.

### Study Objectives

#### Phase 1

1. To identify the current knowledge base of health care professionals within the community setting, regarding the management of UI in women
2. To identify treatment strategies and referral pathways available to women with UI in the community setting
3. To explore any variation in the management of athletic women with UI compared to that for all women

#### Phase 2

To determine the following:

1. Recruitment rate: the ease with which athletic women with UI may be recruited directly from sports clubs and gyms
2. Eligibility rate: the proportion of volunteers screened that are eligible for inclusion
3. Consent rate: the proportion of those eligible women that consent both for the trial and, separately, for intimate examination of their pelvic floor muscles
4. Attendance rate: the proportion of participants who attend over 50% of planned sessions
5. Data completion rate: the number of questionnaires returned at the 3-month and 6-month time points

#### Phase 3

To establish:

1. Acceptability of the recruitment process
2. Acceptability of the intervention to participants
3. Outcome measures: to identify the acceptability of the outcome measures to participants for use in a future study
4. Choice of venue: acceptability of venue to the participants
5. Ease of use and perceived benefit of using a smartphone ‘app’ to aid PFMT adherence and recording
6. Acceptability to participants of being randomised for treatment in a future trial

A traffic light system (based on that used in a protocol by Pitt et al. 2020) [27] will be used to review progression criteria to a full trial. Green will indicate that it is feasible to progress to a definitive trial with only minor or no changes made to the study design and procedures, amber will indicate that modifications should be made before progressing and red will indicate that it is not feasible to progress with this design (see Table 1).

**Table 1:** Table to illustrate criteria for progression from feasibility study to a definitive trial

Progression criteria	Measurement	Green	Amber	Red
**Phase 2**				
Recruitment	Number of participants recruited within 6 months	15–20	10–15	< 10
Eligibility	Proportion of those screened that are eligible	> 75% screened are eligible	Minor changes to eligibility criteria would increase the number to > 75%	Majority of those screened are ineligible or changes to inclusion criteria required would prohibit meaningful results
Initial consent	Proportion of eligible participants who consent	> 70%	50–69%	< 50%
Consent to intimate examination	Proportion of those enrolled who consent to intimate examination of PFM	> 70%	50–69 %	< 50%
Attendance	Number of scheduled appointments attended by participants	> 75%	50–75%	< 50%
Data completion	Follow-up questionnaire collected at 3-month review	> 75%	50–75%	< 50%
	Follow-up questionnaires collected at 6-month review	> 60%	30–60%	<30%
**Phase 3**				
Recruitment process	Qualitative process evaluation	Most participants find the recruitment process acceptable or minor changes requested	Participants views on acceptability conflicting or larger changes required	Most participants find the recruitment process unacceptable or the changes required are unrealistic
Acceptability of intervention	Qualitative process evaluation	Most participants find the intervention acceptable or would request only minor alterations	Views on acceptability conflicting or major revisions needed	Most participants find the intervention unacceptable or changes required are not feasible
Acceptability of outcome measures	Qualitative process evaluation	Most participants find the questionnaires acceptable or would request only minor alterations	Views on acceptability conflicting or major revisions needed	Most participants find the questionnaires unacceptable or changes required are not feasible
Choice of venue	Qualitative process evaluation	Most participants find the venue acceptable or would request only minor alterations	Views on acceptability conflicting or major revisions needed	Most participants find the venue unacceptable or changes required are not feasible
Use of Squeezy App	Qualitative process evaluation	Most participants find use of a smartphone app easy and beneficial as a reminder for PFMT	Fewer than half find use of a smartphone app beneficial	Most participants find use of a smartphone app not helpful or easy to use
Acceptability of being randomised in a future trial	Qualitative process evaluation	Most participants would accept being randomised for interventions in a future trial	Most would accept being randomised for interventions if there was an option to receive the intervention post RCT	Most participants would not accept being part of a control group in an RCT

This table has been adapted from Pitt et al. 2020 [27]

The steering group will oversee the final decision on whether to progress to a definitive study dependent on the results.

### Study Design

This is a mixed methods study with three distinct but related phases.

#### Phase 1

##### Aim

To identify current first-line treatment provision within the community for UI in women and specifically athletic women. This is to establish current practice in order to inform the control arm of a future trial.

##### Participants

We will recruit six to eight local health care professionals (HCP) from the Derbyshire and Nottinghamshire areas for interview. This has been dictated largely by the time and resources available but will also provide a snapshot of the key issues to be addressed in future research. The HCP will be comprised of GPs, nurses, and physiotherapists as these are likely to be the point of first contact for women with UI in the community. We will aim to recruit the same numbers from all three professions. The aim is to recruit professionals that woman seek help from in the first instance, rather than to specifically recruit those with a particular specialisation in pelvic or women’s health. All participants will be required to provide full, informed, written consent before any data is collected.

##### Interviews

These semi-structured interviews will explore the current general understanding of what constitutes appropriate management of UI in the community. It will establish whether participants are aware of NICE guidelines or if there is any local pathway for the management of UI or a specific referral process. Further, it will explore if the HCP would feel confident to provide structured rehabilitation for the PFM and whether they would supervise this or whether they would seek referral to a specialist physiotherapist or a continence advisor. We will ascertain whether they would manage athletic or sporting women in the same way as other women with UI and if they feel that women with UI should be encouraged to continue with their sport despite the incontinence. Participants will be asked to discuss their understanding of specialist pelvic health physiotherapy, what it entails and if they know where and/or how they could refer to such a service. These interviews will be conducted by a specialist pelvic health physiotherapist with an understanding of the pathways and local services. Where interviewees do not raise particular points, for example, knowledge of the appropriate NICE guidelines, they will be prompted about this using a pre-prepared interview schedule.

##### Setting

Interviews will take place at a time and in a place that is acceptable to the participants and are planned to last for up to 30 min.

#### Phase 2

##### Aims

To investigate the feasibility of recruitment and retention of athletic and/or sporting women who self-report the symptoms of SUI, UUI, MUI and/or increased urinary urgency and/or frequency, from the community for a trial of physiotherapeutic management of UI. Further, to explore the proportion of those screened who are eligible and then consent to the process and to an intimate examination. To record the attendance rates and data completion at 3 and 6 months

##### Consent

All participants will be required to provide full, informed, written consent before any data is collected and the intervention begins. In addition, participants in phase 2 will receive further information regarding having a digital vaginal examination (DVE) of their pelvic floor muscles. In line with Chartered Society of Physiotherapy (CSP) guidelines, the option of a chaperone will be offered for this procedure should participants wish [28]. Further informed written consent will be required prior to the DVE. If any participant does not wish to proceed at this stage, they will be given a leaflet and general advice and will be offered the opportunity to continue with the questionnaires at 3 months and at 6 months. This will provide an indication of potential attrition of participants not wishing to receive specialised physiotherapy, for a future trial.

##### Participants

We will recruit 15–20 sporting and/or athletic women from local sports clubs and gyms who self-report symptoms of UI.

All participants must be female athletes or regular exercisers, 18 years of age and over, who self-report symptoms of UI.

UI will be defined as leaking of urine associated with increased abdominal pressure such as impact, coughing and/or sneezing, leaking associated with urinary urgency and will include increased urinary urgency and/or urinary frequency such that it is bothersome to the woman [2].

Athletic or sporting women will be defined as adult women exercising (moderate to vigorous) or participating in sport three or more times a week and for more than 150 min (i.e. meeting or exceeding The UK Chief Medical Officers’ Physical Activity Guidelines [29]).

Participants will not be eligible if they are new to sport within the last year, are pregnant or less than 1 year post-natal, or have commenced oestrogen or anticholinergic treatment within the previous 3 months. They will be ineligible if they are involved in ongoing physiotherapy/continence advice treatment elsewhere within the previous year or have an existing neurological condition that may contribute to UI. Participants that are unable to read or understand English will be ineligible for the study as the study documents are in English.

##### Recruitment

We will target gyms and sports clubs in the local community via social media, email and flyers to identify potentially interested members. Managers of the gyms and secretaries of the sports clubs will be offered the opportunity to host informative talks by the researcher (KGC) regarding pelvic health issues in women and provision of further information regarding the study.

Interested participants who make contact by email or by telephone will be sent participant information sheets. They will be directed to reply by email acknowledging their interest in proceeding within the study and providing a telephone number and time for the researcher to contact them: this will be taken as consent for that telephone number to be used for this purpose. Within the follow-up telephone call, any further questions the participant may have will be answered, they will be screened for eligibility, and an appointment will be booked for those that wish to take part.

##### Intervention

At the initial appointment, the study regimen and questionnaires will be explained in more detail. In addition, participants will be informed how to complete the logs to record their weekly sporting activities and fluid charts that will be required as part of the trial. Further, they will be issued with two jugs to enable accurate measurement of input and output of fluid/urine and a pot to collect a mid-stream urine sample (MSU), in order to check for any signs of urinary tract infection, as recommended in the NICE guidelines [11]. Clinically we have found that patients have often misunderstood how to complete 3-day fluid charts correctly if these are not explained face-to-face. This meeting will also allow time for a full explanation of the assessment and treatment process, including the digital vaginal examination of their pelvic floor muscles, and whether they might wish to have a chaperone for this procedure. It is good practice ( Chartered Society of Physiotherapy) to ensure that all patients are given full information regarding any intimate examination prior to this appointment [28]. Face-to-face contact allows for explanations and time for the participant to consider fully whether they wish to proceed. Written informed consent will be taken prior to any data collection. A second appointment will then be made where baseline questionnaires, the fluid chart, the MSU and sporting log will be collected. There will be a subjective assessment to explore medical and demographic history and an objective assessment which will include visual inspection and digital palpation of the perineum, vagina and PFM. This is to establish baseline levels of PFM resting tone, power and endurance in crook lying. It is planned to use electromyography (EMG) to monitor resting tone, power and endurance in standing. This will be dependent on the individual participant’s agreement.

Results of the assessments will be recorded and discussed with the participant. A treatment plan, derived from the assessment findings, will be discussed and agreed before proceeding.

The intervention will be tailored to the individual but will involve the core components of pelvic floor muscle training (PFMT). This will have two aspects, a home programme and supervised work within the clinic to ensure correct technique and appropriate progression. PFMT is defined as ‘exercise to improve PFM strength, endurance, power, relaxation or a combination of these parameters’ [30]. In addition, in some cases, the intervention may include soft tissue techniques to release tight muscles where required to comply with the PFMT.

Participants will be taught correct PFM contraction and relaxation, which will be confirmed by digital vaginal examination (DVE) in crook lying. This can be consolidated in standing using EMG biofeedback. As part of the progression, women will be encouraged to practice exercises in different, functional positions. They will be taught to recruit the PFM pre-emptively, to brace before impact (e.g. coughing and sneezing), which is a technique referred to as ‘the knack’ [31].

Participants will be offered access to the ‘Squeezy App’, which is a smartphone app that enables the participant to set reminders for their PFMT. This can be customised for each individual. The ‘Squeezy App’ is connected to an online platform ‘Living With’ where the practitioner can view exercise adherence, as long as the participant uses the app for each set of exercises throughout the day and saves a record to their phone. Those that do not wish to use this app will be provided with a paper log in order that they can record each exercise set within the day, manually.

It is planned that participants will attend for no more than seven appointments, including the initial appointment for explanation and consent, with a specialist pelvic health physiotherapist over a 6-month period. This is in keeping with practice nationally: A recent survey of specialist pelvic health physiotherapists showed that the number of one to one exercise sessions offered was 4.4 ± 1.3 within the NHS [32]. Each appointment will be planned to take 45–60 min, although this may reduce to 30–45 min after the initial assessments are complete. The regularity and number of appointments will depend on the participant’s needs and necessary progression in the programme. Each participant’s individual intervention will be recorded, and information from this will inform future interventions offered as part of a future trial.

##### Setting

Participants will be offered a choice of venue between a community physiotherapy clinic and a private room within the University of Nottingham. Offering a choice of venues will enable us to assess the venues for a future trial in order to optimise participant retention.

##### Outcome measures

As this is a feasibility study, a range of outcome measures will be explored. This will be with a view to defining acceptability for the participants whilst still achieving the optimum data required for the analysis. With this in mind, two questionnaires will be used for participants in phase 2 alongside measures of pelvic floor strength and a record of urinary symptoms:

- The short urinary distress inventory UDI-6: This is a brief measure of how bothersome the symptoms of UI are to the individual. It is easy and quick to complete and is a reliable outcome measure [33].
- The International Consultation on Incontinence Modular Questionnaire Female Lower Urinary Tract Symptoms Long Form Module (ICIQ-FLUTS-lf) [34]: Although this is a long questionnaire, the decision to include this was due to the comprehensive amount of information it reveals. It reports not just the severity and impact of any UI symptoms but also the information about the nature of the urinary symptoms experienced and the effects of these on quality of life. The ICIQ modules are widely used as an outcome measure both in pelvic health research and in clinical practice and have been validated as reliable measures [35].
- A 3-day fluid chart: This will record, throughout, volumes and types of fluid drunk, volumes and frequency of urinary voids and any leaks experienced.
- The objective examination will include a DVE of the participant’s pelvic floor. The resting ‘tone’ of the muscles will be noted within the participant’s notes on a chart of the superficial and deep pelvic floor muscle fibres as low, moderate, or high. Maximum voluntary contraction will be recorded using the modified Oxford scale for the average score over three, 3 s holds with 10 s rest. The amount of PFM movement will also be noted with the contraction, to ensure an upward and inward lift of the perineum [36].
- EMG (electromyogram), as a secondary outcome measure: Where acceptable to the participant a vaginal surface EMG probe, Periform^R^ +, neenpelvichealth.com, will be used to collect data on PFM activity in standing via a Neurotrac Myoplus Pro, https://veritymedical.co.uk/, as commonly used in clinical practice. Measurements will be taken at rest, in standing, to record levels of resting tone and maximum voluntary contraction (MVC). MVC will be the average of three, 3 s maximal holds with 10 s rest. Resting tone will again be noted after maximal efforts to ensure full relaxation post contraction. Although some recent reviews, e.g. Nunes et al. 2019 have suggested that EMG biofeedback offers no real benefit for the treatment of UI over other treatment interventions and PFMT, this could be due to the lack of homogeneity of the studies included. Moreover, EMG provides a valuable addition in terms of motivation for patients who use this tool [37].

The DVE and EMG measurements will be taken after requesting additional consent from the participant.

**Fig. 1:**
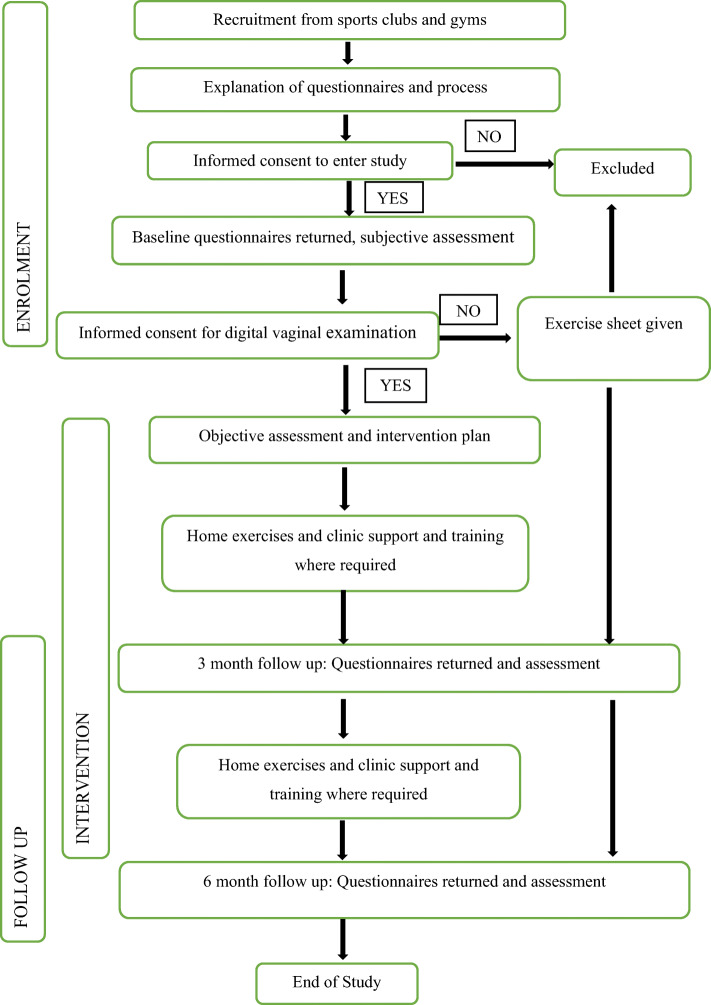
Flow chart of participants’ progress through phase 2

Each outcome, including the objective assessments, will be assessed and recorded at baseline, at 3 months and at 6 months after recruitment. It is planned to collect the outcome measures at scheduled appointments. The fluid charts, sporting logs, UDI 6 and ICIQ-FLUTS-lf will be in the form of paper questionnaires. The data from UDI 6 and ICIQ-FLUTS-lf will not be analysed until the study is concluded. Should participants not be able or willing to attend, an email will be sent requesting completion of the questionnaires. A stamped-addressed envelope will be provided for return. See Fig. 1 for details of flow of participants in the study.

#### Phase 3

##### Aim

To investigate the acceptability to participants of the recruitment process, the intervention, the outcome measures and the venue. It will collect data regarding participants’ experiences of using the smartphone app and their thoughts regarding randomisation in a future study. We will also explore previous treatments for UI that participants may have accessed and the effects of UI on their quality of life and on their sporting activity specifically.

##### Participants

Six to eight participants from phase 2 will be recruited for an in-depth interview. This will be a purposeful selection to ensure a range of ages, types of incontinence and sporting backgrounds. Participants will be required to provide additional informed, written consent before any data is collected for this phase. This is again in keeping with the time and resources available to us.

##### Interviews

Semi-structured interviews will explore the acceptability of the recruitment process, the outcome measures, the setting and the intervention. It is also planned that the interviews will gain further insight into the impact of UI on the participants’ quality of life and their sporting aspirations. The interviews in this phase will be conducted by an experienced qualitative researcher who has not been previously involved in phase 1 or 2. We will refer to the theoretical framework of acceptability when devising the interview schedule in phase 3 in order that the data provides richer information regarding the intervention and acceptability of the study design [38].

##### Setting

Interviews will be conducted at a time and place convenient to the participant and are expected to take approximately 30 min.

#### Sample size and justification

In phases 1 and 3, we plan to recruit six to eight participants. This reflects the time and resources available for these phases of the study. This sample size has been determined as being of sufficient size to address the research aims within the time frame and funding available [39].

In phase 2, we will recruit 15–20 athletic or sporting women. As this is a feasibility study, the purpose is to obtain estimates of recruitment and retention rates alongside the acceptability of the intervention. Therefore, a formal sample size calculation is not required. Traditionally, sample sizes of between 24 and 50 are recommended for a feasibility study, but the intensity and length of the proposed intervention also needs to be considered [40]. We believe that this sample size is sufficient to identify attrition, eligibility, consent, attendance and data completion rates, within the time frame available to the study.

#### Analysis

In phases 1 and 3, digital audio-recordings of the interviews will be transferred to a secure and password-protected file on a dedicated web server at the University of Nottingham. Personal identifiers will be removed. The recording will then be deleted from the recording device. Anonymised recordings will be sent electronically to a transcribing service approved by the University of Nottingham and then uploaded into the qualitative software  package NVivo 12 for line by line coding. Qualitative data from these recordings will be analysed using a framework approach [41], which is a pragmatic method of organising, analysing and interpreting data around focal research questions. Anonymised interview data will be read and re-read several times, to explore data for themes before coding begins. Three researchers will each review a sample of the transcriptions in order to identify themes across the datasets. A working analytical framework will be developed, and data chunks will be transferred onto a framework matrix. Thematic table summaries will be used to generate recommendations about the nature of the subsequent trial; specific detail will also be used to inform recruitment strategies, data collection regimes and participant information resources.

In phase 2, numbers and characteristics of participants will be summarised using descriptive statistics, and completeness of data will be assessed. Descriptive summaries of outcome data at each follow-up time point will be presented. The Template for Intervention, Description and Replication (TIDieR) guidelines will be used to describe the details of the intervention to ensure that it is reported in sufficient detail for replication [42]. As previously described, a traffic light system will be used to identify whether each outcome has met the requirements for progression of this study to a definitive trial.

#### Participant Withdrawal

In each of the study phases, participants may withdraw from the study either at their own request or be withdrawn at the discretion of the Investigator. Participants will be made aware (via the participant information sheet and consent form) that should they withdraw, the data collected cannot be erased and may still be used in the final analysis.

Participants who withdraw will be replaced if this is possible within the time frame of the study.

#### Data management

Each participant will be assigned a study identity code number, for use on study forms, other study documents and the electronic database. Study forms shall be restricted to those personnel approved by the Chief Investigator and recorded as such in the study records.

Medical records will be stored securely and in accordance with good clinical practice for a time period of 8 years from the last intervention. This is the recommended time for record retention by the Chartered Society of Physiotherapy who will be providing professional liability. Computer-held data including the study database will be held securely and password protected. All data will be stored on a secure dedicated web server.

#### Adverse events

The occurrence of an adverse event as a result of participation within this study is not expected in phases 1 or 3. Any adverse event data will be collected and reported.

It is again unlikely that any adverse events are likely to arise as part of the intervention in phase 2, as the intervention is standard physiotherapy practice for this issue, but any observation of unexpected pathology or trauma, e.g. cancer, female genital mutilation or history of abuse during the subjective or objective parts of the assessment process, will be discussed with the gynaecologist advising the research team, and appropriate advice taken.

## Discussion

UI is a debilitating condition that affects up to 40% of all UK women [1] and up to a third of young nulliparous female athletes [16]. Despite this, there is little evidence regarding the effects of treatment in this population and there is need for high-quality RCTs to investigate the benefits and cost effectiveness of PFMT to treat PFM disorders in athletes [21]. This article provides the methodology for an initial study investigating the feasibility of conducting a future RCT of physiotherapy management of urinary incontinence in athletic women.

We anticipate several potential challenges within the study:

1. As we have no means of knowing the number of potential participants our flyers will reach, we are unable to estimate the denominator for recruitment. We can, however, collate potential issues with recruitment in this environment, such as the ease of distribution within clubs and gyms, and explore ways to potentially maximise distribution.
2. The subject of UI is a sensitive area and it may be that potential participants are reluctant to present due to embarrassment or that they may not wish to share fully their experiences with us in phase 3.
3. We plan to use a smartphone app to help illustrate compliance. Whilst feedback from users and clinicians has shown that this improves compliance and outcomes, we do not know whether it is effective at illustrating compliance as it will still rely on the user documenting the incidence of their exercises effectively.
4. The acceptability of the intervention will be assessed post intervention, and some have argued that this may not reflect whether a healthcare intervention is truly acceptable [43]. However, by giving a full explanation by telephone and then again in person before embarking on the full assessment, we will allow volunteers to select this intervention as acceptable or not at this point.

By employing a mixed methodology, we will be able to obtain information on current knowledge and services from a number of sources. Further, it will maximise the collection of important data in order to inform a potential future trial.

## Trial status

The trial was registered on clinicaltrials.gov (NCT03986411) on 14 June 2019. Recruitment of participants was initiated on 17 September 2019 and will conclude on 31 March 2020. We have currently recruited and interviewed seven of eight possible participants in phase 1 and 10 of 20 potential participants in phase 2.

## Dissemination and outputs

The study results will be shared via publication in peer-reviewed academic journals, will be disseminated at conferences and will be deposited in the University of Nottingham, Research Information Service Repository for access.

## Data Availability

The datasets used and/or analysed during the current study will be available from the corresponding author on reasonable request and in accordance with consent and ethical approval.

## Abbreviations

UI: Urinary incontinence
SUI: Stress urinary incontinence
UUI: Urgency-related urinary incontinence
MUI: Mixed urinary incontinence
PFM: Pelvic floor muscles
HCP: Health care professional
RCT: Randomised controlled trial
PFMT: PFM training
DVE: Digital vaginal examination
EMG: Electromyogram
UDI 6: Short form urinary distress inventory
ICIQ-FLUTS-lf: International Consultation on Incontinence Questionnaire Female Lower Urinary Tract Symptoms long form
MVC: Maximum voluntary contraction
MSU: Mid-stream urine sample
